# Experimental validation of a recently developed model for single-fiber reflectance spectroscopy

**DOI:** 10.1117/1.JBO.26.2.025004

**Published:** 2021-02-27

**Authors:** Anouk L. Post, Dirk J. Faber, Henricus J. C. M. Sterenborg, Ton G. van Leeuwen

**Affiliations:** a University of Amsterdam, Amsterdam UMC, Cancer Center Amsterdam, Amsterdam Cardiovascular Sciences, Department of Biomedical Engineering and Physics, Amsterdam, The Netherlands; b The Netherlands Cancer Institute, Department of Surgery, Amsterdam, The Netherlands

**Keywords:** single fiber reflectance spectroscopy, reflectance spectroscopy, subdiffuse

## Abstract

**Significance:** We recently developed a model for the reflectance measured with (multi-diameter) single-fiber reflectance (SFR) spectroscopy as a function of the reduced scattering coefficient $\mu_{s}^{\prime }$, the absorption coefficient $\mu_{a}$, and the phase function parameter $p_{\mathrm{sb}}$. We validated this model with simulations.

**Aim:** We validate our model experimentally. To prevent overfitting, we investigate the wavelength-dependence of $p_{\mathrm{sb}}$ and propose a parametrization with only three parameters. We also investigate whether this parametrization enables measurements with a single fiber, as opposed to multiple fibers used in multi-diameter SFR (MDSFR).

**Approach:** We validate our model on 16 phantoms with two concentrations of Intralipid-20% ($\mu_{s}^{\prime }=13$ and $21\;{\mathrm{cm}}^{-1}$ at 500 nm) and eight concentrations of Evans Blue ($\mu_{a}=1$ to $20\;{\mathrm{cm}}^{-1}$ at 605 nm). We parametrize $p_{\mathrm{sb}}$ as $10^{-5}\cdot (p_{1}(\lambda /650)+p_{2}{\left(\lambda /650\right)}^{2}+p_{3}{\left(\lambda /650\right)}^{3})$.

**Results:** Average errors were 7% for $\mu_{s}^{\prime }$, 11% for $\mu_{a}$, and 16% with the parametrization of $p_{\mathrm{sb}}$; and 7%, 17%, and 16%, respectively, without. The parametrization of $p_{\mathrm{sb}}$ improved the fit speed 25 times (94 s to <4  s). Average errors for only one fiber were 50%, 33%, and 186%, respectively.

**Conclusions:** Our recently developed model provides accurate results for MDSFR measurements but not for a single fiber. The $p_{\mathrm{sb}}$ parametrization prevents overfitting and speeds up the fit.

## 1 Introduction

Reflectance spectroscopy techniques are used to determine optical properties of tissue and relate these to various types of disease. In single-fiber reflectance (SFR) spectroscopy light is emitted and collected through the same fiber, connected to a broadband light source and a spectrograph to detect the reflectance versus wavelength. Compared to diffuse reflectance techniques, the sampling depth of SFR spectroscopy is smaller, in the order of a few hundred micrometers.^1^ This small sampling depth makes SFR spectroscopy suitable to detect small-scale, superficial changes, such as changes related to early-stage or epithelial cancers. Since SFR fibers generally have a diameter of a few hundred micrometers, they can be introduced through endoscopes or biopsy needles, which enables the use of SFR spectroscopy in internal organs such as the colon or esophagus. SFR spectroscopy has mainly been studied for medical applications in the field of oncology,^2^^–^^9^ but also in saturation monitoring,^10^ orthopedics,^11^^,^^12^ and to examine melanin in the skin.^13^

SFR measurements cannot be described by diffusion theory alone. The diffusion approximation can accurately describe the reflectance for distances between photon emission and collection of more than several transport mean free paths ($1/\mu_{s}^{\prime }$). For smaller source–detector separations, measurements are in the subdiffuse regime, where the measured reflectance is sensitive to the tissue phase function [the probability distribution of scattering angles, p(θ)].^14^^–^^16^ Until recently, only the model of Kanick et al.^1^^,^^17^ was available to extract optical properties from SFR measurements. The validity of their model was limited to tissues with modified Henyey–Greenstein (MHG) phase functions. However, for many tissue types, it has been shown that the MHG phase function underestimates scattering in the backward direction.^18^^–^^24^ Therefore, we recently developed a model that is valid for the wide range of phase functions that can be encountered in tissue.^25^^–^^27^ In this model, the reflectance is a function of the fiber diameter d, the reduced scattering coefficient $\mu_{s}^{\prime }$, the absorption coefficient $\mu_{a}$, and the phase function parameter $p_{\mathrm{sb}}$. Our model was validated with Monte Carlo (MC) simulations. Here, we validate our model experimentally on phantoms with varying concentrations of Intralipid-20% and Evans Blue by fitting our model to the measured spectra.

For robust and accurate fit results, it is important that the model is not overfitting the data—a fit model should consist of the minimum number of parameters required to describe the data. To minimize the number of fit parameters, the reduced scattering coefficient is generally parameterized as $\mu_{s}^{\prime }=a\cdot {\left(\lambda /\lambda_{0}\right)}^{-b}$, where a is the scattering amplitude, b is the scattering slope, and $\lambda_{0}$ is a reference wavelength, and the absorption coefficient is parameterized as the sum of absorption spectra [$\mu_{a}(\lambda )$] of different absorbers present in the tissue and the volume fraction ($\varphi_{i}$) of these absorbers. However, the wavelength-dependence of $p_{\mathrm{sb}}$ is currently not known and, therefore, a separate fit parameter for $p_{\mathrm{sb}}$ has to be used for each wavelength, resulting in a few hundred fit parameters. A fit of our model to a spectrum measured by a single fiber would provide a non-unique solution because the number of fit parameters would be larger than the number of data points since a separate value of $p_{\mathrm{sb}}$ is estimated for each wavelength and the reflectance also depends on the reduced scattering and absorption coefficient. A solution to reduce the number of fit parameters compared to the number of data points is performing measurements with two different fiber diameters—also called multi-diameter SFR (MDSFR).^28^ In MDSFR, a fit is performed on the spectra of both fibers simultaneously, assuming they interrogate a sample volume with the same optical properties. Thus in MDSFR, the number of fit parameters stays the same as in SFR, but the number of data points is doubled. Even so, without a parametrization of $p_{\mathrm{sb}}$ we are most likely still overfitting our data. To prevent overfitting, we need to parametrize the wavelength-dependence of $p_{\mathrm{sb}}$ by the smallest number of parameters possible—decreasing the number of fit parameters from a few hundred to just a few. A parametrization of $p_{\mathrm{sb}}$ might also enable measurements with only a single fiber.

Previously, we validated our model based on MC simulations. In this paper, we validate our model experimentally on phantoms. Furthermore, we propose a parametrization of $p_{\mathrm{sb}}$ and compare fit results for our model with and without the use of this parametrization of $p_{\mathrm{sb}}$. Finally, we investigate whether the $p_{\mathrm{sb}}$ parametrization could enable measurements with only a single fiber.

## 2 Background

We recently developed a model for the reflectance measured by SFR spectroscopy as a function of the fiber diameter d, the reduced scattering coefficient $\mu_{s}^{\prime }$, the absorption coefficient $\mu_{a}$, and the phase function parameter $p_{\mathrm{sb}}$. The full derivation can be found in Refs. 25–27. In short, we modeled the reflectance as the sum of a semiballistic reflectance ($R_{\mathrm{SFR},\mathrm{sb}}$) and a diffuse reflectance ($R_{\mathrm{SFR},\mathrm{dif}}$):

$$R_{\mathrm{SFR}}=R_{\mathrm{SFR},\mathrm{sb}}+R_{\mathrm{SFR},\mathrm{dif}}=(1+X)\cdot R_{\mathrm{SFR},\mathrm{dif}},$$ (1)

where X is the ratio between the semiballistic and diffuse reflectance. The diffuse reflectance $R_{\mathrm{SFR},\mathrm{dif}}$ equals the collection efficiency of the fiber ($\eta_{c}$) times the fraction of photons that are diffuse and reach the fiber face ($R_{\mathrm{dif}}$):^26^

$$R_{\mathrm{SFR},\mathrm{dif}}=\eta_{c}\cdot R_{\mathrm{dif}}=1.11\cdot {\left(\frac{\mathrm{NA}}{n}\right)}^{2}\cdot R_{\mathrm{dif}},$$ (2)

where NA is the fiber numerical aperture and n is the tissue refractive index. The fraction of photons that are diffuse and reach the fiber face ($R_{\mathrm{dif}}$) is calculated as^27^

$$R_{\mathrm{dif}}(\mu_{s}^{\prime }d,\mu_{a}d)=\frac{\pi }{4}\cdot d^{2}\cdot \overset{d}{\underset{0}{\int }}R(\rho ,\mu_{s}^{\prime },\mu_{a})\cdot p(\rho ,d)d\rho ,$$ (3)

where $R(\rho ,\mu_{s}^{\prime },\mu_{a})$ is the diffuse reflectance as a function of radial distance (ρ) for a pencil beam illumination using the extended boundary condition as proposed by Farrell et al.,^29^

$$R(\rho ,\mu_{s}^{\prime },\mu_{a})=\frac{a^{\prime }}{4\pi }[z_{0}(\mu_{\mathrm{eff}}+\frac{1}{r_{1}})\frac{e^{-\mu_{\mathrm{eff}}\cdot r_{1}}}{r_{1}^{2}}+(z_{0}+2z_{b})(\mu_{\mathrm{eff}}+\frac{1}{r_{2}})\frac{e^{-\mu_{\mathrm{eff}}\cdot r_{2}}}{r_{2}^{2}}],$$ (4)

where $a^{\prime }=\mu_{s}^{\prime }/(\mu_{s}^{\prime }+\mu_{a})$; $z_{0}=1/(\mu_{s}^{\prime })$; $\mu_{\mathrm{eff}}=\surd (3\mu_{a}\mu_{s}^{\prime })$; $r_{1}=\surd (z_{0}^{2}+\rho^{2})$ and $r_{2}=\surd ((z_{0}+2z_{b}{)}^{2}+\rho^{2})$; $z_{b}=2A/(3\mu_{s}^{\prime })$; and A is a parameter that depends on the refractive index mismatch between the fiber and the tissue, which is equal to 1.027 for a fiber refractive index of 1.45 and a tissue refractive index of 1.35.^30^ The integral of the diffuse reflectance versus radial distance is performed over the probability density function of distances over the fiber face p(ρ,d), which is a classic problem in the field of geometric probability:^31^

$$p(\rho ,d)=\frac{16\rho }{\pi d^{2}}\cos^{-1}(\frac{\rho }{d})-\frac{16}{\pi d}{\left(\frac{\rho }{d}\right)}^{2}\sqrt{1-{\left(\frac{\rho }{d}\right)}^{2}}.$$ (5)

To incorporate the influence of the phase function on semiballistic photons, we developed the parameter $p_{\mathrm{sb}}$, which we defined as^25^

$$p_{\mathrm{sb}}=\frac{p_{b}(1\;\deg)}{1-p_{f}(23\;\deg)},$$ (6)

where $p_{b}$ (1 deg) is the integral of the phase function over 1 deg in the backward direction and $p_{f}$ (23 deg) is the integral over 23 deg in the forward direction. We modeled the ratio X between semiballistic and diffuse photons in the absence of absorption as^25^

$$X_{0}=\;\frac{R_{\mathrm{SFR},\mathrm{sb}}}{R_{\mathrm{SFR},\mathrm{dif}}}=c_{1}{\left(\frac{p_{\mathrm{sb}}}{{\left(\mu_{s}^{\prime }d\right)}^{2}}\right)}^{c_{2}}.$$ (7)

For low values of $\mu_{s}^{\prime }d$, the diffuse reflectance scales with ${\left(\mu_{s}^{\prime }d\right)}^{2}$, which is why we included that term in the denominator of Eq. (7), and we assumed that the semiballistic contribution scales differently with $\mu_{s}^{\prime }d$, which is accounted for through the parameter $c_{2}$. To include the influence of absorption, the reflectance can be written as the product of the reflectance in the absence of absorption and the integral of the photon path length distribution p(l) weighted by the Beer–Lambert law:

$$R(\mu_{a})=R_{0}\cdot \overset{\infty }{\underset{0}{\int }}p(l)e^{-\mu_{a}l}dl.$$ (8)

Equation (8) has the form of a Laplace transform, where the absorption coefficient and pathlength are conjugate variables. According to the scaling properties of the Laplace transform, the diffuse reflectance thus depends on the ratio $\mu_{a}/\mu_{s}^{\prime }$. Absorption only has a minor influence on semiballistic photons due to shorter path lengths. Therefore, we included the term $\mu_{a}/\mu_{s}^{\prime }$ into our model to incorporate the influence of absorption:^26^

$$X=\;\frac{R_{\mathrm{SFR},\mathrm{sb}}}{R_{\mathrm{SFR},\mathrm{dif}}}=c_{1}{\left(\frac{p_{\mathrm{sb}}}{{\left(\mu_{s}^{\prime }d\right)}^{2}}\right)}^{c_{2}}\cdot e^{c_{3}\cdot {\left(\frac{\mu_{a}}{\mu_{s}^{\prime }}\right)}^{c_{4}}}.$$ (9)

The constants were derived based on over 10, 000 MC simulations: $c_{1}=3046$; $c_{2}=0.748$; $c_{3}=1.17$; $c_{4}=0.57$.^25^^,^^26^

## 3 Methods

### 3.1 Parametrization of Tissue *p*_sb_

To develop a parametrization of $p_{\mathrm{sb}}$, we investigated the wavelength-dependent behavior of $p_{\mathrm{sb}}$ by modeling tissue as a combination of discrete particles. Based on Mie theory, we calculated the phase function and $p_{\mathrm{sb}}$ versus wavelength. Gélébart et al.^32^ first proposed to model tissue as a fractal distribution of scattering spheres. Since then, several studies have shown that this model can accurately represent measured tissue optical properties.^33^^,^^34^ In the fractal model, the number density ($\rho_{N}$) of particles with a diameter $D_{\text{par}}$ is described by

$$\rho_{N}(D_{\mathrm{par}})={D_{\mathrm{par}}}^{-f},$$ (10)

where f is the fractal dimension. We used the MATLAB code of Mätzler^35^ to determine the differential scattering cross-section ($\sigma_{s}$) versus scattering angle of a single particle diameter, which depends on the size parameter x and the refractive index ratio m:

$$x=\;\frac{\pi D}{\lambda n_{\mathrm{med}}},$$ (11)

$$m=\;\frac{n_{\mathrm{par}}}{n_{\mathrm{med}}},$$ (12)

where $n_{\text{med}}$ and $n_{\text{par}}$ are the refractive indices of the medium and particle, respectively. The phase function for the entire size distribution is the normalized sum of the differential scattering cross sections weighted by the volume fraction of scattering particles per diameter $f_{v}(D_{i})$.

$$p(\theta )=\;\frac{\sum_{i=1}^{n}f_{v}(D_{i})\cdot \sigma_{s}(\theta ,D_{i})}{\sum_{i=1}^{n}f_{v}(D_{i})\cdot \sigma_{s}(D_{i})}.$$ (13)

Based on the paper from Wang,^34^ we used 1.354 as the medium refractive index and 1.42 as the particle refractive index. We calculated the phase function using a discretized size distribution with particle diameters from 5 to 6000 nm, equally spaced on a logarithmic scale in 100 steps. We determined the Mie phase functions and $p_{\mathrm{sb}}$ values over the wavelength range of 400 to 900 nm, in steps of 5 nm.

Two types of phase functions that have been measured in tissue are the MHG and the two-term Henyey–Greenstein (TTHG). We modeled 6 size distributions, to match 3 MHG and 3 TTHG phase functions at 635 nm based on the discrete particle model (Table 1). We used 635 nm since most phase function measurements have been performed at 632 or 635 nm. We performed a fit to obtain the fractal dimension f that would result in the best match between the obtained [Eq. (13)] and the desired phase function (Table 1). However, no particle size distributions obeying Eq. (10) could provide a good match with the MHG and TTHG phase functions. Therefore, we first performed a fit using Eq. (10) to obtain a single value for the parameter f that would result in a phase function close to the desired phase function. Next, we performed a second fit using Eq. (14), where a value for the parameter $f_{\text{par}}$ was obtained for each particle diameter $D_{\text{par}}$.

$$\rho_{N}(D_{\mathrm{par}})=D_{\mathrm{par}}^{-f_{\mathrm{par}}}.$$ (14)

For each particle diameter, $f_{\text{par}}$ was allowed to deviate 5% from f. Without a restriction, the fit was unstable. The limit of 5% was chosen since a smaller limit (1% to 2%) would not result in a phase function that accurately represented an MHG or TTHG phase function and a larger limit (>10%) would result in a size distribution that did not resemble a fractal distribution anymore. For the obtained size distributions, the phase functions and $p_{\mathrm{sb}}$ spectra were calculated.

**Table 1:** Parameters describing the phase functions used in our analysis. The MHG equals $p_{\text{MHG}}=\alpha \cdot p_{\mathrm{HG}}(\theta )+(1-\alpha )3/4\pi \;\cos^{2}(\theta )$ and the TTHG equals $p_{\mathrm{TTHG}}=\alpha \cdot p_{\mathrm{HG}}(g_{f},\theta )+(1-\alpha )\cdot p_{\mathrm{HG}}(g_{b},\theta )$, where $p_{\mathrm{HG}}$ is the HG phase function; $g_{1}$ is the scattering anisotropy.

phase function type	α	$g_{b}$	$g_{f}$	$g_{HG}$	$g_{1}$
MHG	0.98	—	—	0.90	0.90
	0.97	—	—	0.87	0.85
	0.96	—	—	0.83	0.80
TTHG	0.91	−0.15	0.84	—	0.76
	0.92	−0.21	0.9	—	0.83
	0.92	−0.29	0.85	—	0.77

### 3.2 Experimental Validation

#### 3.2.1 MDSFR device

**Fig. 1:**
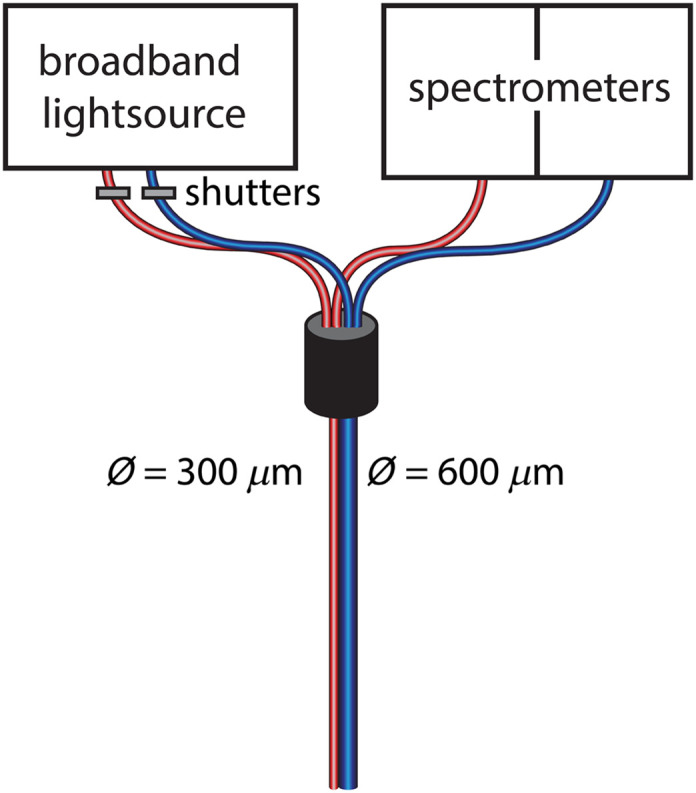
(MD)SFR setup. Two fibers with diameters of 300 and 600  μm, respectively, and NAs of 0.22 were used for the measurements. Shutters were placed to enable separate illumination by each measurement fiber and each fiber was connected to a separate spectrometer. Figure reproduced from Ref. 9.

Measurements were performed with a custom-made MDSFR device (Fig. 1). A fiber with a 300-μm core and a fiber with a 600-μm core (Optran WF, Diamond Kimberlit B.V., Almere, the Netherlands) with numerical apertures of 0.22 were connected to a halogen light source (Ocean Optics, HL-2000-FHSA) using a bifurcated fiber. The tips of the measurement fibers were polished at an angle of 15 deg, to minimize internal reflection from the fiber tip. Shutters were placed to enable separate illumination by each measurement fiber. Each fiber was connected to a separate spectrometer (Avantes ADC1000-USB). Data acquisition was performed using a custom-written LabVIEW program and analyzed using a custom-written Matlab program.

#### 3.2.2 Phantoms

We prepared two sets of phantoms with Intralipid-20% (Fresenius Kabi) and varying concentrations of Evans Blue (Sigma-Aldrich). One set was diluted with water to an Intralipid-20% volume fraction of 1:16, the second set to a volume fraction of 1:25, resulting in $\mu_{s}^{\prime }$ values between approximately 7 and $25\;{\mathrm{cm}}^{-1}$ over the full spectrum. Even though higher $\mu_{s}^{\prime }$ values can occur in tissue, we had to stay at these lower volume fractions to prevent dependent scattering within the Intralipid-20% dilutions since for dependent scattering the phase function can currently not be predicted and, therefore, we would not know what the value of $p_{\mathrm{sb}}$ would be to compare our results to. We prepared an Evans Blue stock solution of 5 g/l and determined its absorption spectrum using a transmission measurement through 1 cm of the stock solution diluted to a volume fraction of 0.4:80.4. Based on the absorption spectrum, we prepared samples with Intralipid 20% and Evans Blue to obtain $\mu_{a}$ = [1.0; 2.0; 3.1; 5.1; 7.7; 10.2; 15.1; 20.1] ${\mathrm{cm}}^{-1}$ at 605 nm (the peak of the measured absorption spectrum). We chose these values for the absorption coefficient since, in tissue, the maximum absorption coefficient of blood between 500 and 600 nm is expected to be between 3 and $15\;{\mathrm{cm}}^{-1}$, which corresponds to blood volume fractions of 1% to 5%.

We compared the obtained values for $\mu_{s}^{\prime }$ to the measured $\mu_{s}^{\prime }$ of Michels et al.^36^. We calculated reference values for $p_{\mathrm{sb}}$ using Mie theory and the size distribution for Intralipid-20% as supplied by Michels et al.:

$$\rho_{N}(D_{\mathrm{par}})=10^{-f_{\mathrm{IL}}},$$ (15)

where $f_{\mathrm{IL}}$ is equal to $4.{151\cdot 10}^{3}{\mathrm{nm}}^{-1}$.^36^ It is noteworthy that the size distribution for Intralipid-20% is not a fractal distribution. Based on this size distribution, we calculated the phase function for a discrete distribution of 10 diameters, from 25 to 750 nm, in steps of 50 nm. Following the approach of Michels et al., we used the wavelength-dependent refractive index of water for $n_{\mathrm{med}}$ and the wavelength-dependent refractive index of soy oil for $n_{\mathrm{par}}$ using the Cauchy equation:

$$n(\lambda )=I+J\lambda^{-2}+K\lambda^{-4},$$ (16)

with $I_{\text{water}}=1.311$, $I_{\text{soy}}=1.451$ and the same values of J and K for both water and soy oil of $J=1.{154\cdot 10}^{4}$ and $K=-1.{132\cdot 10}^{9}$.^36^^,^^37^

#### 3.2.3 Data analysis

The number of counts obtained from the spectrometer was corrected for the nonlinearity of the detector.^38^ Next, the number of counts ($I_{\text{sample}}$) was converted to an absolute reflectance using:

$$R_{\mathrm{sample}}(\lambda )=\frac{I_{\mathrm{sample}}(\lambda )-I_{\mathrm{back}(\lambda )}}{I_{\mathrm{ref}}(\lambda )-I_{\mathrm{back}(\lambda )}}R_{\mathrm{ref}}(\lambda ),$$ (17)

where $I_{\mathrm{back}}$ was a measurement performed in a black container with water, to include both the dark current of the spectrometer and internal reflections at the fiber tip. $I_{\text{ref}}$ was a measurement performed on Intralipid-20% diluted with water to a volume fraction of 1:20. $R_{\text{ref}}$ was the absolute reflectance of the 1:20 Intralipid dilution, obtained using the Fresnel reflection method of Zhang et al.^39^

The measured spectra were fitted using the model of Post et al.^25^^,^^26^ and minimizing the chi-squared value of the fit. In Eq. (2), we set the refractive index to 1.33 (the refractive index of water), which makes A equal to 1.0311 for Eq. (4). To reduce the number of fit parameters, $\mu_{s}^{\prime }$ was modeled as $\mu_{s}^{\prime }=a{\left(\lambda /\lambda_{0}\right)}^{-b}$, with $\lambda_{0}=500\;\mathrm{nm}$, and $\mu_{a}$ was modeled as the product of the volume fraction of Evans Blue ($\varphi_{\mathrm{EB}}$) and the absorption spectrum of Evans Blue obtained from the transmission measurement ($\mu_{a,\mathrm{EB}}$).

In MDSFR, the spectra measured by both fibers are fitted simultaneously, resulting in a single set of optical properties for both fibers. To investigate the influence of the parametrization of $p_{\mathrm{sb}}$ on the fit results with two fibers, the analysis was performed once using the parametrization of $p_{\mathrm{sb}}$ and once where a value of $p_{\mathrm{sb}}$ was fitted per wavelength. First, the measurements with both fiber diameters were fitted simultaneously. Next, we investigated whether it was possible to accurately extract optical properties from a measurement with only a single fiber, using the parametrization of $p_{\mathrm{sb}}$. For each fit, we calculated the confidence intervals based on the method proposed by Amelink et al.^40^

## 4 Results

### 4.1 Parametrization of Tissue p_sb_

**Fig. 2:**
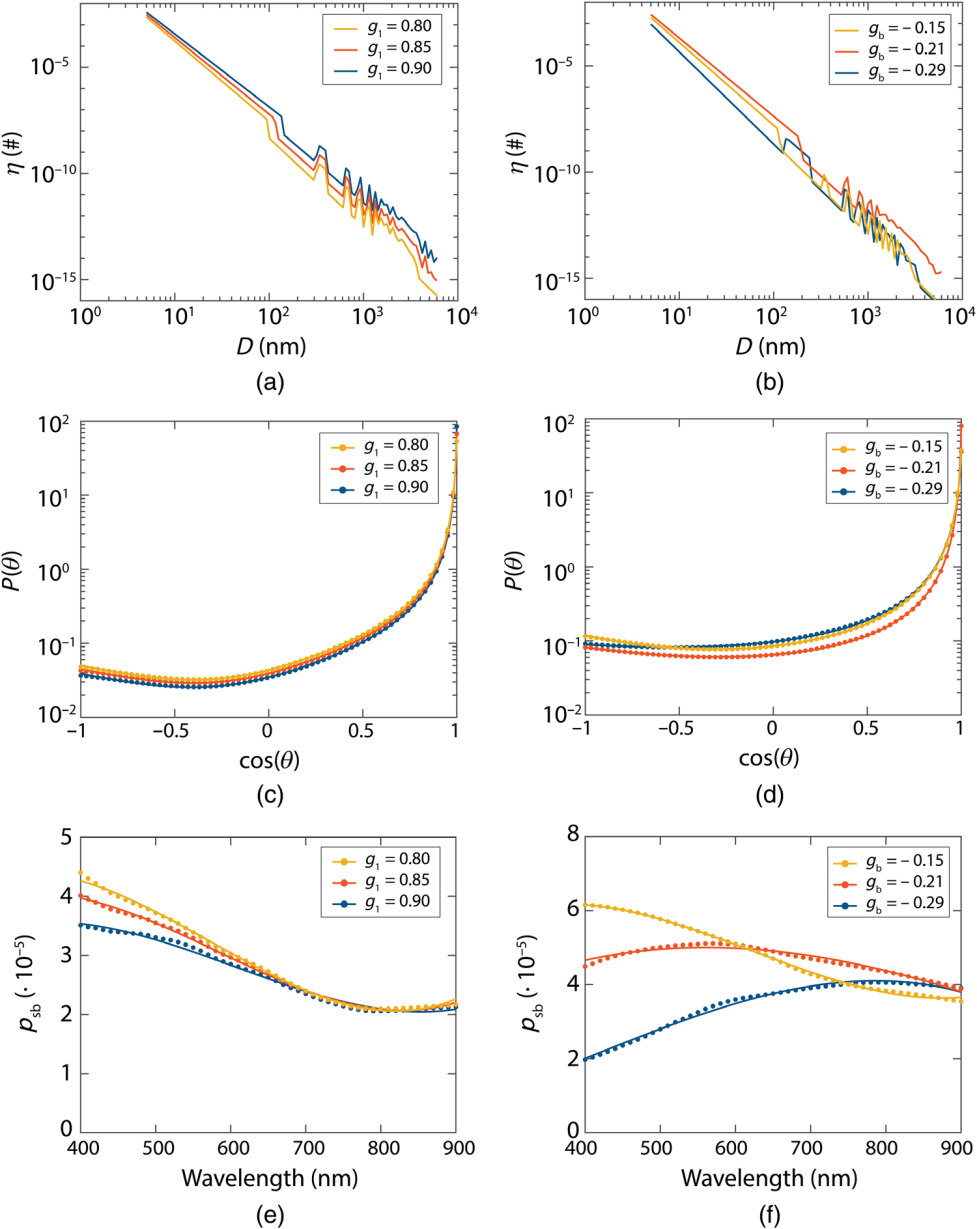
Results for MHG (left) and TTHG (right) phase functions. (a)–(b) size distributions used for each phase function; (c)–(d) phase functions obtained with these size distributions (dots) and desired phase functions (lines); (e)–(f) resulting $p_{\mathrm{sb}}$ values (dots) and fit results with Eq. (18) (lines).

Figure 2 displays the six different size distributions [Figs. 2(a) and 2(b)], their corresponding phase functions [Figs. 2(c) and 2(d)], and $p_{\mathrm{sb}}$ spectra [Figs. 2(e) and 2(f)]. All six phase functions were accurately represented by the used size distributions, the average difference between each obtained and desired phase function (Table 1) was 0.6% to 1.8%. Compared to a true fractal distribution, the size distributions all had lower frequencies of larger sphere sizes. All the $p_{\mathrm{sb}}$ spectra are smooth functions and could be accurately represented by third-order polynomials. The average difference between the third-order polynomials and $p_{\mathrm{sb}}$ values was 0.4% to 1.0%. Lower order polynomials were less accurate, e.g., for a second-order polynomial, the average difference was 1.4% to 3.9%. To further reduce the number of fit parameters, we investigated whether we need the full third-order polynomial. We found that removing the constant offset term had only a minor influence on the accuracy—the average difference between that parametrization and $p_{\mathrm{sb}}$ was 0.6% to 1.7%. Thus, we parametrize $p_{\mathrm{sb}}$ with only three parameters as

$$p_{\mathrm{sb}}=10^{-5}\cdot (p_{1}(\lambda /650)+p_{2}{\left(\lambda /650\right)}^{2}+p_{3}{\left(\lambda /650\right)}^{3}).$$ (18)

We normalized the wavelength at 650 nm since that is the middle of the obtained spectrum (400 to 900 nm). Normalizing the wavelength in the middle of the spectrum ensures the smallest differences in $p_{1},p_{2}$, and $p_{3}$ values over the spectrum, which will lead to more stable fit results. The factor $10^{-5}$ was chosen based on the fact that $p_{\mathrm{sb}}$ ranges from $10^{-6}$ to $10^{-4}$ in tissue.^25^

### 4.2 Phantoms

**Fig. 3:**
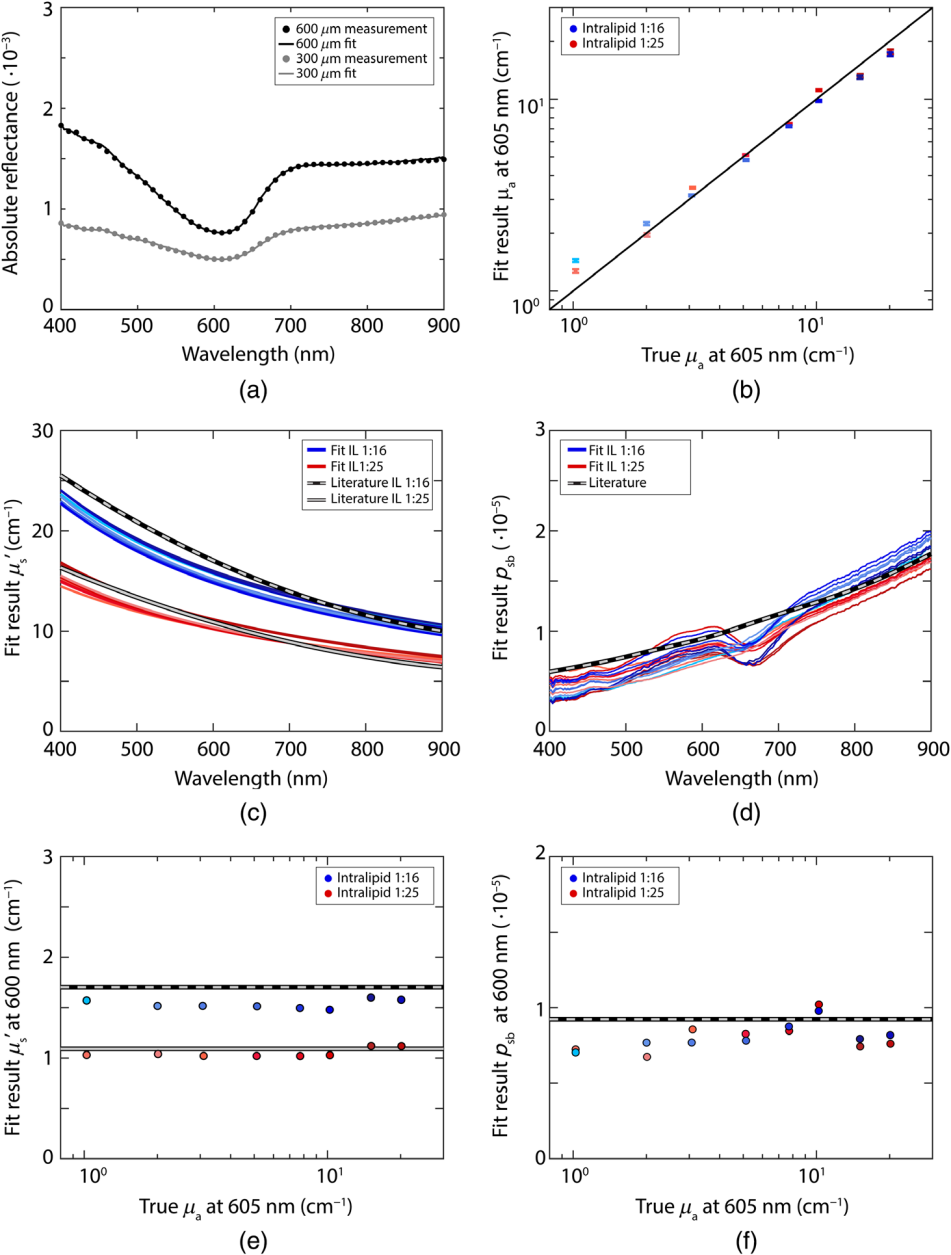
Results of the fit procedure without a parametrization of $p_{\mathrm{sb}}$, for measurements with both a 300-μm fiber and a 600-μm fiber. (a) Example fit of the absolute reflectance, every 10 data points of the measurement are plotted to improve visibility; (b) fit results for the absorption coefficient at 605 nm for phantoms with Intralipid 20% diluted 1:16 (blue) and 1:25 (red), error bars indicate the confidence intervals of the fit; (c) fit results for the reduced scattering coefficient, where the colors go from light to dark with increasing Evans Blue concentration; (d) fit results for $p_{\mathrm{sb}}$; (e) fit results for the reduced scattering coefficient at 600 nm; (f) fit results for $p_{\mathrm{sb}}$ at 600 nm.

**Fig. 4:**
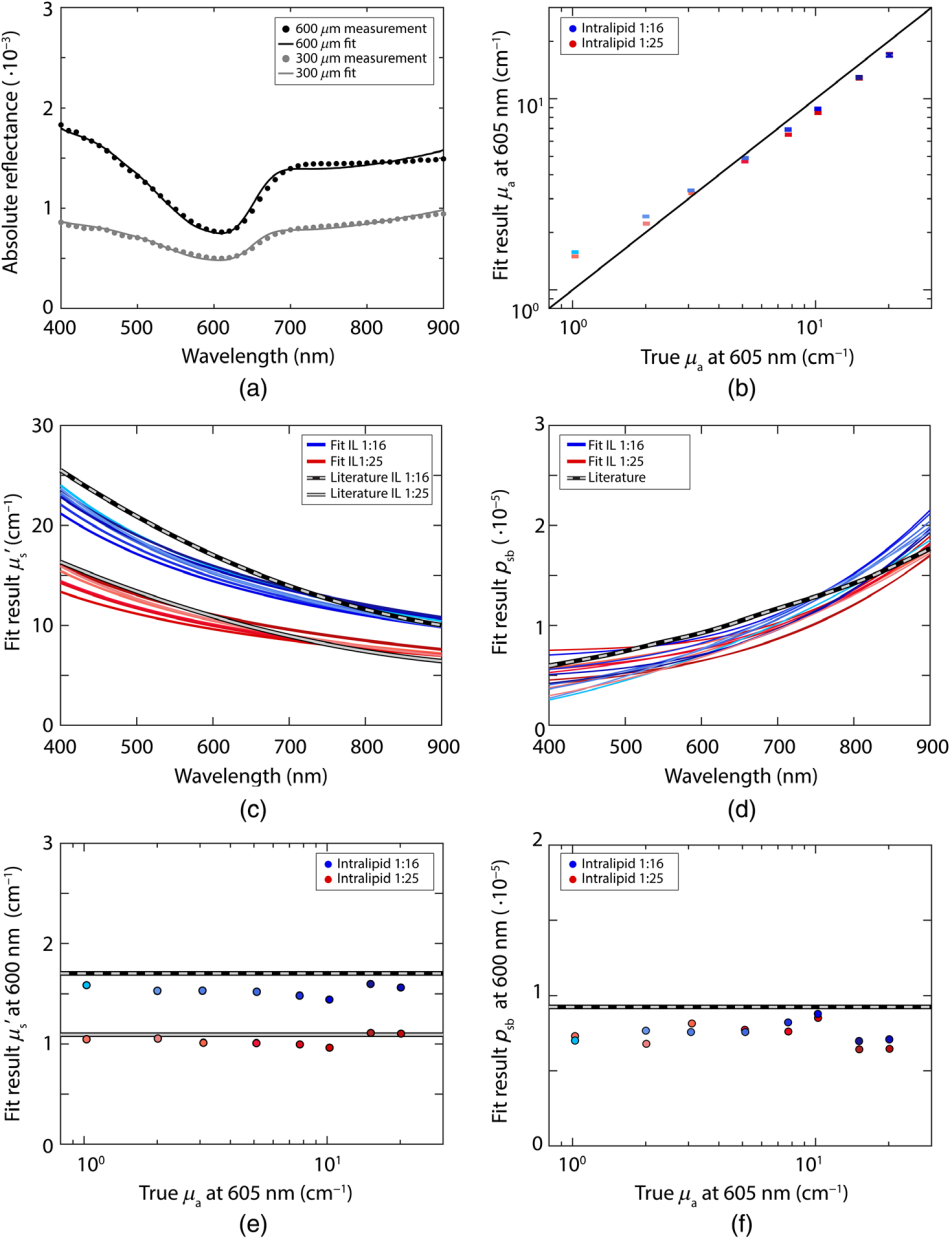
Results of the fit procedure with a parametrization of $p_{\mathrm{sb}}$ [Eq. (18)], for measurements with both a 300-μm fiber and a 600-μm fiber. (a) Example fit of the absolute reflectance, every 10 data points of the measurement are plotted to improve visibility; (b) fit results for the absorption coefficient at 605 nm for phantoms with Intralipid-20% diluted 1:16 (blue) and 1:25 (red); error bars indicate the confidence intervals of the fit; (c) fit results for the reduced scattering coefficient, where the colors go from light to dark with increasing Evans Blue concentration; (d) fit results for $p_{\mathrm{sb}}$; (e) fit results for the reduced scattering coefficient at 600 nm; (f) fit results for $p_{\mathrm{sb}}$ at 600 nm.

Figure 3 depicts the fit results without using a parametrization of $p_{\mathrm{sb}}$ (a separate value for $p_{\mathrm{sb}}$ is estimated for each wavelength) and Fig. 4 depicts the fit results while using Eq. (18) for $p_{\mathrm{sb}}$. The results for the reduced scattering coefficient are similar between performing the fit with and without a $p_{\mathrm{sb}}$ parametrization; the mean error in the reduced scattering coefficient averaged over all samples and wavelengths was 7% both with and without the $p_{\mathrm{sb}}$ parametrization. The results for the absorption coefficient are slightly better without using a parametrization for $p_{\mathrm{sb}}$; the mean error in the concentration averaged over all samples and wavelengths was 17% with the $p_{\mathrm{sb}}$ parametrization and 11% without the parametrization. For $p_{\mathrm{sb}}$ itself, the mean error over all samples and wavelengths was 16% both with and without the $p_{\mathrm{sb}}$ parametrization. With the $p_{\mathrm{sb}}$ parametrization, the confidence intervals for the reduced scattering coefficient and $p_{\mathrm{sb}}$ were smaller but the confidence intervals for the absorption coefficient were larger. The signature of the absorption spectrum of Evans Blue is present in the fit result for $p_{\mathrm{sb}}$ when no parametrization for $p_{\mathrm{sb}}$ is used. On average, a fit with the $p_{\mathrm{sb}}$ parametrization was 25 times faster (>4  s), compared to a fit without the parametrization (94 s).

**Fig. 5:**
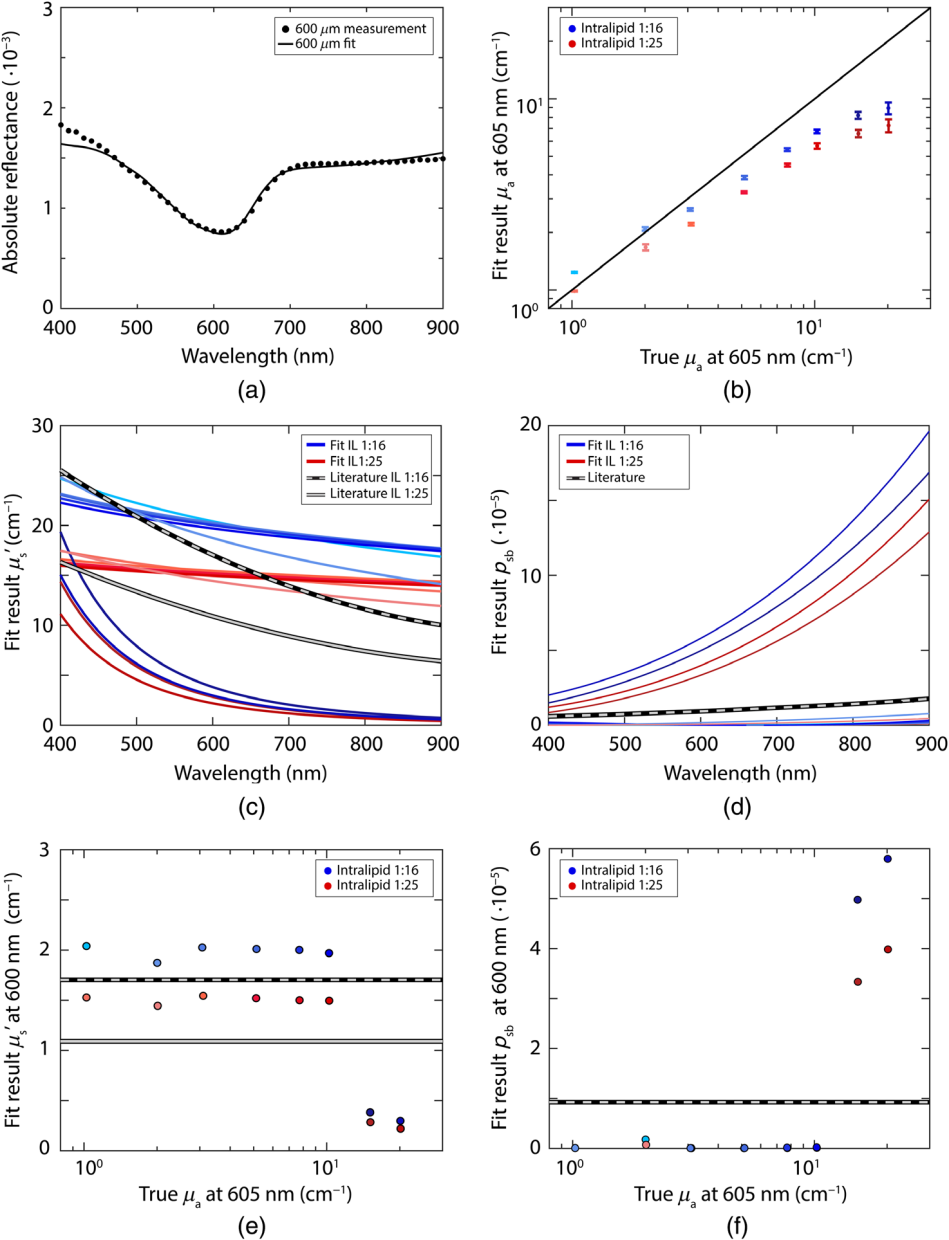
Results of the fit procedure with a parametrization of $p_{\mathrm{sb}}$ [Eq. (18)] using only the data of the 600  μm fiber. (a) Example fit of the absolute reflectance, every 10 data points of the measurement are plotted to increase visibility; (b) fit results for the absorption coefficient at 605 nm for phantoms with Intralipid 20% diluted 1:16 (blue) and 1:25 (red); error bars indicate the confidence intervals of the fit; (c) fit results for the reduced scattering coefficient, where the colors go from dark to light with increasing Evans Blue concentration; (d) fit results for $p_{\mathrm{sb}}$. It is worthy to note the different vertical axis in (d) compared to Figs. 3 and 4. (e) Fit results for the reduced scattering coefficient at 600 nm. (f) Fit results for $p_{\mathrm{sb}}$ at 600 nm. For the concentrations with a $\mu_{a}$ of $1\;{\mathrm{cm}}^{-1}$ and from 3.1 to $10.2\;{\mathrm{cm}}^{-1}$ the $p_{\mathrm{sb}}$ values at 600 nm obtained for both Intralipid concentrations overlap.

Figure 5 shows the result of performing a fit on the reflectance values of a single fiber of 600  μm, using the $p_{\mathrm{sb}}$ parametrization. Compared to fitting reflectance values from both fibers, the error in the absorption coefficient is 2 to 3 times as high (average error of 33%), the error in the reduced scattering coefficient is 7 times as high (average error of 50%), and the error in $p_{\mathrm{sb}}$ is much worse (average error of 185%).

## 5 Discussion

We validated our model for the reflectance as a function of tissue optical properties experimentally on phantoms of Intralipid-20% and Evans Blue. For the measurements with two fibers, the extracted optical properties were accurate, validating our model and the used calibration procedure. The absorption coefficient was overestimated below approximately $5\;{\mathrm{cm}}^{-1}$ and underestimated above approximately $8\;{\mathrm{cm}}^{-1}$. The underestimation for higher absorption coefficients is in line with our previous results, where we concluded that $\mu_{a}d$ values above 4 would result in less accurate results,^26^ which corresponds to an absorption coefficient of $6.7\;{\mathrm{cm}}^{-1}$ for a fiber diameter of 600  μm. For low values of $\mu_{a}$, the influence of absorption on the reflectance is minimal, which could explain the inaccuracies at lower absorption coefficients.

We developed a parametrization of $p_{\mathrm{sb}}$ to prevent overfitting. Incorporating the parametrization of $p_{\mathrm{sb}}$ into the fit did not affect the average error in the estimated scattering coefficient and $p_{\mathrm{sb}}$ but increased the average error in the estimated absorption coefficient from 11% to 17%. Without a parametrization of $p_{\mathrm{sb}}$, the fit perfectly matches the data, but the absorption signature of Evans Blue is seen in the $p_{\mathrm{sb}}$ fit result. This cross-talk between absorption and $p_{\mathrm{sb}}$ is most likely a sign of overfitting. When a separate value of $p_{\mathrm{sb}}$ is fitted for each wavelength, the values of $p_{\mathrm{sb}}$ can be adapted to perfectly match the reflectance values to compensate for errors in the model for the reflectance as a function of tissue optical properties or errors in the measurement.

To prevent overfitting, a fit model needs to include the minimum number of parameters necessary to describe the data. Therefore, we parametrized the wavelength-dependence of $p_{\mathrm{sb}}$ with the smallest number of parameters possible, which is 3. Our previously developed model for the reflectance as a function of the tissue optical properties $\mu_{s}^{\prime }$, $\mu_{a}$, and $p_{\mathrm{sb}}$ was based on physical principles. We know that the reflectance in the subdiffuse regime depends on $\mu_{s}^{\prime }$, $\mu_{a}$, and the tissue phase function and we have captured the influence of the phase function in a single parameter, $p_{\mathrm{sb}}$. Since we know that $\mu_{s}^{\prime }$, $\mu_{a}$, and $p_{\mathrm{sb}}$ are necessary to describe the reflectance for (MD)SFR spectroscopy, and we parameterized these optical properties with the minimum number of parameters required to describe them, we feel confident that our model combined with the parametrization of $p_{\mathrm{sb}}$ does not result in overfitting of the measured spectra and we, therefore, recommend using Eq. (18) to parametrize $p_{\mathrm{sb}}$. Furthermore, another advantage of parametrizing $p_{\mathrm{sb}}$ is that it speeds up the fit by a factor of 25. For most clinical applications, a fit procedure that takes 1.5 min per spectrum is not acceptable.

We hypothesized that using a parametrization of $p_{\mathrm{sb}}$ would enable SFR measurements with only a single fiber. However, the fit results for only a single fiber were worse compared to two fibers, with average errors of 33% for $\mu_{a}$, 50% for $\mu_{s}^{\prime }$, and 186% for $p_{\mathrm{sb}}$. A possible explanation for these results is that $\mu_{s}^{\prime }$ and $p_{\mathrm{sb}}$ compete in the fit when only a single fiber is used since they have a similar wavelength-dependence. Even though $\mu_{s}^{\prime }$ and $p_{\mathrm{sb}}$ were estimated inaccurately, the fit matched the measured reflectance well. This demonstrates that different combinations of $\mu_{s}^{\prime }$ and $p_{\mathrm{sb}}$ can result in the same spectra when only a single fiber is used. Based on these results, we do not recommend performing SFR measurements with only a single fiber, because even though the fit to the reflectance can seem accurate, the extracted optical properties cannot be trusted. Even though MDSFR measurements with two fibers increase the probe size compared to a single fiber, MDSFR still has a small footprint compared to DRS. SFR fibers generally have a diameter in the order of a few hundred micrometers, making an MDSFR probe smaller than a millimeter, facilitating introduction through biopsy needles or working channels of endoscopes. Furthermore, since the sampling depth of MDSFR is much smaller than DRS (a few hundred micrometers versus a few millimeters), MDSFR is more suitable to detect superficial changes in tissue such as the development of epithelial cancers. Furthermore, MDSFR is more sensitive than DRS to changes in the tissue phase function, providing additional information that could prove useful for the detection of disease.^2^^,^^3^^,^^9^

For the parametrization of $p_{\mathrm{sb}}$, we investigated its wavelength-dependence using Mie theory and different size distributions of scattering particles for MHG and TTHG phase functions. Some other phase functions have been proposed for tissue, such as the modified power of cosines^16^ or the Reynolds McCormick (RMC, also known as Gegenbauer kernel).^24^ Our selection of phase functions was based on publications where the phase function of tissue had been measured. Also, we only included types of phase functions that matched well with the measured phase functions. For example, in early papers, the Henyey–Greenstein (HG) phase function was often used, but looking at the data, the HG did not fit the measured phase function well—which is why later the MHG and TTHG were proposed. The MHG phase function has been measured in the skin^41^ and the majority of measured tissue phase functions have been TTHG phase functions.^18^^–^^24^ To the best of our knowledge, the only other type of phase function that has been used in a paper, where the phase function was measured, was the RMC. In that paper, the RMC phase function was proposed for measurements of full blood.^23^ For blood, the RMC describes the forward-directed scattering accurately, but still greatly underestimates the backward-directed scattering, which will result in an underestimation of $p_{\mathrm{sb}}$. Therefore, we did not include RMC phase functions in our analysis.

MHG and TTHG phase functions could not be produced using a pure fractal distribution. The necessity to deviate from a pure fractal distribution to match MHG and TTHG phase functions might be the result of non-spherical scatterers within the tissue, scatterers with a different refractive index than the assumed refractive index of 1.42 and/or simply that the true size distribution of scattering particles in tissue is not necessarily a pure fractal distribution. We investigated TTHG phase functions with $g_{b}\geq -0.3$. Lower values of $g_{b}$ have been measured, e.g., −0.54 and −0.56 in the human lung and uterus,^18^ but we could not reproduce them using Mie theory, not even when we searched for a random size distribution of particles. This could be explained by the fact that these higher $g_{b}$ values occur in the regime where dependent scattering occurs. Dependent scattering is the interference of scattering by particles that are closely packed together, which results in relatively more backscattering. Currently, it is not possible to include the effect of dependent scattering on the phase function using Mie theory for polydisperse spheres. Nevertheless, we do expect that using a third-order polynomial will provide accurate results for most—if not all—tissue phase functions. Phase functions are the result of the spatial distribution of refractive index fluctuations compared to the wavelength. For a monodisperse sample, the phase function will change quite rapidly with wavelength, but not for polydisperse samples. Since tissue can be modeled as a polydisperse sample, we expect that the tissue phase function, and thus $p_{\mathrm{sb}}$, will still change gradually with wavelength. If any phase functions other than the MHG and TTHG are measured for tissue samples in the future, this hypothesis can be tested.

## 6 Conclusion

In conclusion, optical properties can be accurately extracted using our recently developed model for the reflectance measured with MDSFR as a function of optical properties. A third-order polynomial without the offset term can be used to parametrize the wavelength-dependence of $p_{\mathrm{sb}}$, which prevents overfitting and dramatically improves the speed of the fitting procedure. Finally, optical properties cannot be determined accurately by SFR measurements with only a single fiber.

## Code, Data, and Materials Availability

The code and data are available upon request.
